# Impact of water solvation on the charge carrier dynamics of organic heterojunction photocatalyst nanoparticle dispersions†

**DOI:** 10.1039/d4sc04030a

**Published:** 2024-10-21

**Authors:** Soranyel Gonzalez-Carrero, Jan Kosco, Teng Fei, Iain McCulloch, James R. Durrant

**Affiliations:** a Department of Chemistry and Centre for Processable Electronics, Imperial College London London W12 0BZ UK soranyel.gonzalez@uv.es j.durrant@imperial.ac.uk; b Institute of Molecular Science, University of Valencia 46980 Paterna Valencia Spain; c King Abdullah University of Science and Technology (KAUST), KAUST Solar Center (KSC) Thuwal 23955-6900 Saudi Arabia iain.mcculloch@chem.ox.ac.uk; d Department of Chemistry, Chemistry Research Laboratory, University of Oxford Oxford OX1 3TA UK; e Department of Electrical and Computer Engineering, Andlinger Center for Energy and the Environment, Princeton University Princeton NJ 08544 USA; f SPECIFIC IKC, College of Engineering, Swansea University Bay Campus, Fabian Way, Wales Swansea SA1 8EN UK

## Abstract

Organic heterojunction nanoparticles (NP) have recently gained significant interest as photocatalysts for visible light-driven hydrogen production. Whilst promising photocatalytic efficiencies have been reported for aqueous NP dispersions, the underlying dynamics of photogenerated charges in such organic heterojunction photocatalysts and how these might differ from more widely studied dry heterojunction films remain relatively unexplored. In this study, we combine transient optical spectroscopies over twelve orders of magnitude in time, using pulsed and continuous light illumination, to elucidate the differences in the charge carrier dynamics of heterojunction NP dispersions, dried NP films, and bulk heterojunction films prepared by spin coating. The ultrafast fast (ps to ns) transient absorption results show efficient charge generation and indistinguishable nanosecond charge recombination decay kinetics of separated charges in all three samples. In contrast, on the slower μs to ms time range, the decay kinetics of heterojunction NP dispersion exhibited up to 15-fold larger amplitude and more than one order of magnitude slower decay of the photogenerated charges than those in films. The analysis of the nanomorphology, NP surfactant, polymer residual metal content and local polar environment suggest that the longer lifetime differences (in ms) in the charge recombination in NP dispersion are mostly associated with a charge carrier stabilisation on a shallow density of states on the NP surface of ∼350 meV by interaction with local water environment, resulting in suppressed charge recombination. The lengthening of NP dispersion charge carrier lifetime is discussed regarding the energetic loss for function and their implications in photocatalysis.

## Abstract 2

Organic heterojunction nanoparticles dispersions exhibited remarkably longer charge carrier lifetimes compared to dry films, resulting from charge stabilisation at the nanoparticle surface by exposure to the local water environment.

## Graphical abstract

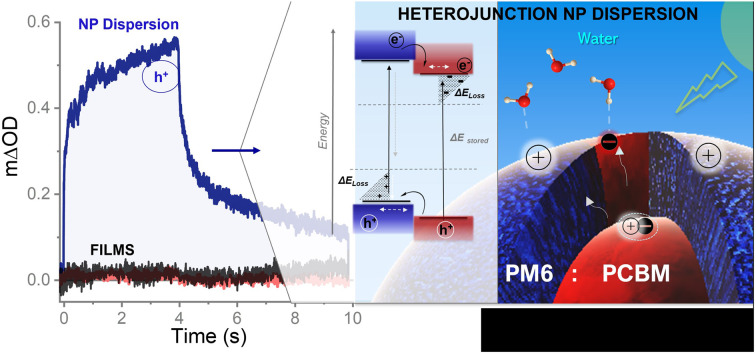

## Introduction

There is increasing interest in photocatalytic pathways to green hydrogen.^1^ Whilst most research to date has focused on inorganic photocatalysts, there is increasing interest in carbon-based materials, including carbon nitride and, most recently, organic semiconductor photocatalysts, such as conjugated polymers and non-fullerene acceptors (NFAs). Organic semiconductors are of particular interest because of their spectral tunability, enabling solar light harvesting across the visible and often near-infrared spectrum. Such organic semiconductors have primarily to date been employed for solar energy conversion in photovoltaic devices, with polymer donor : NFA acceptor bulk heterojunction (BHJ) organic solar cells (OSC) now achieving power energy conversion efficiencies (PCE) exceeding 19%.^2–4^ OSCs have also been fabricated from donor : acceptor (D : A) heterojunction nanoparticles (NP) dispersions, motivated initially as a more sustainable route to device fabrication.^5,6^ Although such NP-processed OSCs exhibit relatively modest efficiencies (up to 11% PCE),^5–8^ such D : A heterojunction NPs have recently gained significant interest in organic photocatalysis. Aqueous organic heterojunction NP dispersions are showing promising efficiencies as photocatalysts for hydrogen production in the presence of sacrificial electron donors, with several studies, including our own, reporting external quantum efficiencies (EQE) up to 10% at wavelengths >600 nm.^9–14^ Functional studies have addressed, for example, the effect of D/A composition ratios, ionic surfactant and polymer side chains on particle nanomorphology and photocatalytic activity, with the aim of enabling further enhancement in NP dispersion quantum efficiencies.

Exciton and charge carrier dynamics are critical to the efficiency of both OSCs and photocatalysts. However, there is currently only limited understanding of how such dynamics compare between these two systems. In organic BHJ's, photogenerated excitons are dominantly separated by charge transfer at the D–A interface, driven by the energetic offset between the donor and the acceptor energy levels. After exciton separation, charges can dissociate into free charges (polarons) or form bound interfacial electron–hole pairs, often referred to as charge transfer states (CT). This charge generation is followed, in a solar cell, by charge transport and extraction to an external circuit, or, in an NP photocatalyst, by charge transfer to the electrolyte or to a co-catalyst for proton reduction to molecular hydrogen.^11^ In both systems, device efficiency is limited by competing exciton decay/charge recombination pathways. It is important to note that charge transport/extraction in OSCs is typically occurring on the 100 ns timescale, *i.e.* several orders of magnitude faster than the kinetics of charge transfer to the electrolyte and proton reduction catalysis in photocatalytic systems. As such, avoiding charge recombination losses limiting device performance is expected to be more challenging for photocatalytic devices than for photovoltaic devices.^15^

In OSCs, much progress has been made in understanding their underlying charge separation, recombination and trapping dynamics, enabling the development of materials design guidelines to optimise device efficiency.^16^ These studies have also helped elucidate the devices' energetic losses and in particular, the energetic cost associated with separating short-lived excitons into charge carriers with sufficient lifetime to enable efficient charge extraction.^15^ A further consideration includes the impact of the disordered nature of most organic semiconductors (intra- or intermolecular, chemical defects, end groups), which can result in shallow trap states extending into the bandgap (*e.g.*, sub-bandgap tails states) impacting significantly on the charge carrier dynamics.^17^

**Fig. 1:**
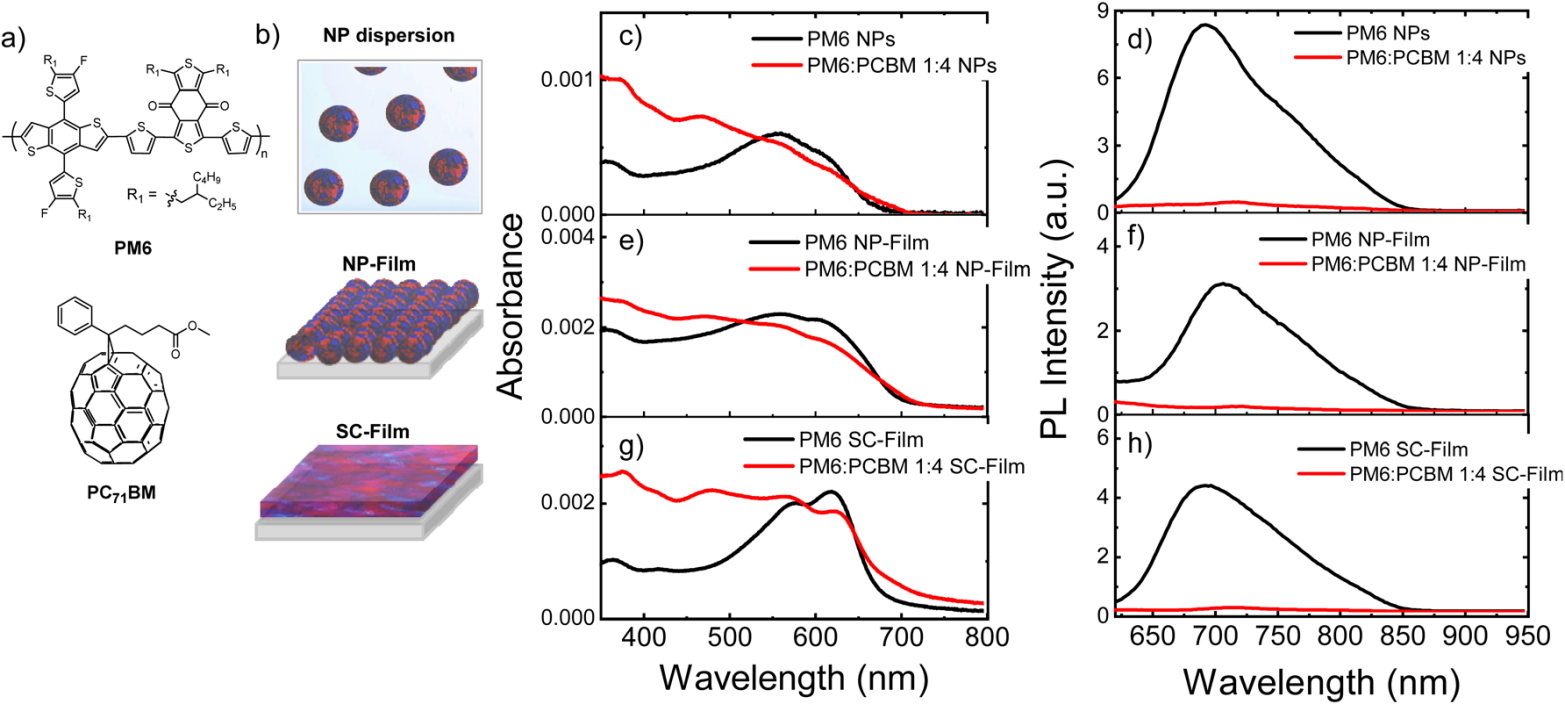
(a) Chemical structures of the organic semiconductors PBDB-T-2F (PM6), and [6,6]-phenyl C71 butyric acid methyl ester (PCBM). (b) Schematic representation of heterojunction NP dispersion, NP-Film and SC-Film. (c–h) Comparison of the absorption and steady-state PL spectra of neat PM6 and PM6 : PCBM 1 : 4 wt% heterojunction in (c and d) NPs dispersion in water; (e and f) NP-film and (g and h) SC-film. The PL spectra were measured at an excitation wavelength of 550 nm.

In D : A heterojunction NPs, photophysical studies have mainly focused on ultrafast timescale (ps to ns), where charge generation occurs,^12,18^ but with only limited studies on charge recombination kinetics and their impact on performance occurring at the slower (μs to ms) timescales when relevant to photocatalysis.^13,19^ We have recently reported a correlation between charge dynamics and the photocatalytic activity of heterojunction NPs of the polymer PBDB-T-2F (PM6) blend with either the fullerene acceptor or NFA, namely [6,6]-phenyl C71 butyric acid methyl ester (PCBM) and BTP-4F (Y6), respectively (Fig. 1). Morphological studies of these NPs showed intermixed or phase-segregated morphologies at high acceptor content, with PM6 : PCBM showing a core/open shell morphology, with PCBM acceptor in the core.^13^ Transient absorption spectroscopy (TAS) indicated that PM6 : Y6 NPs resulted in a higher yield of bound charge pairs (CT states), resulting in fast (ns) geminate recombination, limiting the charge separation and activity. It was suggested that this may result from a more intermixed nanomorphology. We note that another study has reported that efficient charge generation can also be achieved by PM6 : Y6 NPs, most likely associated with differences in NP nanomorphology.^20^ Most strikingly, we observed remarkably long-lived (ms to s) charge accumulation in both heterojunction NP aqueous dispersions. In contrast, no such long-lived charge accumulation could be observed in spin-coated BHJ films prepared with the same materials. The yield of such long-lived photogenerated charges, observed in the absence of an added sacrificial agent, was highest for PM6 : PCBM NPs, correlating with the higher photocatalytic activity for hydrogen evolution of this system.^13^ Another study using time-resolved microwave conductivity also observed a higher accumulation of long-lived charges in heterojunction NP dispersions than in dry heterojunction films.^19^ Both studies indicate that charge carrier dynamics, at least on longer timescales, can substantially differ between NP dispersion and dry heterojunction films, with a potentially critical impact on the ability of these NP dispersions to achieve photocatalytic function.

In the study herein, we focus on the origin and implications of the remarkably long-lived charge accumulation we have observed in heterojunction NPs. We further quantify the energetic cost of this long-lived charge generation, and how this differs from dry BHJ films. Our study focuses on PM6 : PCBM heterojunction NPs, one of the high-performance organic photocatalysts reported to date. Using transient optical spectroscopies ranging from picoseconds to seconds, we contrast the exciton and charge kinetics between PM6 : PCBM heterojunction NPs in aqueous dispersion, the same NPs deposited as a film and then dried and the PM6 : PCBM BHJ thin film deposited from an organic solvent. We then discuss the similarities and differences in kinetics we observe regarding heterojunction morphology, catalytic interactions resulting from residual polymer metals cluster, and the impact of surfactant or water at the NP surface. In particular, we conclude that the remarkably long carrier lifetimes observed for D : A heterojunction NPs in aqueous dispersion predominately result from charge stabilisation by the exposure to high polarity environments (*i.e.* water) at the NP surfaces, enabling increased polarisation and hence charge accumulation, and quantify both the lifetime gain and energetic loss resulting from this stabilisation.

## Results

### Steady-state spectroscopy

Fig. 1 shows the chemical structures of the organic materials explored, and the comparison of the absorption and emission spectra of a colloidal PM6 : PCBM NP dispersion in water prepared by a mini-emulsion method, a thin film fabricated by drop casting of NP dispersion and then drying (NP-Film) and a BHJ film deposited by spin-coating from organic solvent (SC-Film), see preparation details in ESI.† A D : A composition ratio D : A of 1 : 4 wt% was selected for all sample preparations, as we have previously demonstrated that the heterojunction NPs prepared with this stoichiometry (average diameter 80 ± 20 nm) yielded one of the highest hydrogen evolution rate (HER, up to 73.7 mmol h^−1^ g^−1^),^13^ see more NP characterisation details in ESI.†Fig. 1 also shows the spectra of the single polymer PM6 NP dispersion and films (without PCBM) for comparison.

The pristine PM6 NPs exhibited broader and blue-shifted absorption spectra in both NP dispersions and as film (NP-film) compared to SC-film (Fig. 1c, e, and g, black lines). Analogous (but less pronounced) differences were observed in the UV-vis spectra of PM6 : PCBM 1 : 4 wt% heterojunctions NPs and films (Fig. 1, red lines). This result suggests different PM6 morphologies/packing in the SC-Film relative to the NPs. In all three samples, PM6 exciton emission was strongly quenched (>93% at 700 nm) in the heterojunctions relative to neat PM6, indicative of efficient polymer PM6 exciton separation at the D–A interface, and, therefore, of heterojunction formation on a length scale less than the PM6 exciton diffusion length (*i.e.*, 3–15 nm).^4,21,22^

### Charge generation

Ultrafast transient absorption spectroscopy (uf-TAS), in the time range from 500 fs to 6 nanoseconds, was employed to probe the exciton and charge carrier lifetime in neat PM6 and PM6 : PCBM heterojunction NP dispersions, NP-Films and SC-Films. For all three pristine PM6 samples, the uf-TA spectra probed in the NIR (850–1400 nm) showed broad photoinduced absorption with a maximum at 1150 nm, assigned to PM6 singlet exciton absorption (Fig. S1 and S2†).^13,21^ In all the heterojunction samples, the decay dynamics of this PM6 exciton signal were substantially accelerated (by up to 25 times), indicative of efficient electron transfer from PM6 to PCBM (Fig. S3†). Consistent with this conclusion, the accelerated PM6 exciton decay in the heterojunctions is correlated with the appearance of a pronounced, long-lived absorption feature at 950 nm, assigned to the formation of PM6^+^ polarons.^13,23^ These uf-TAS data are, therefore, consistent with our PL quenching data discussed above and confirm efficient exciton separation in all three heterojunction samples.

**Fig. 2:**
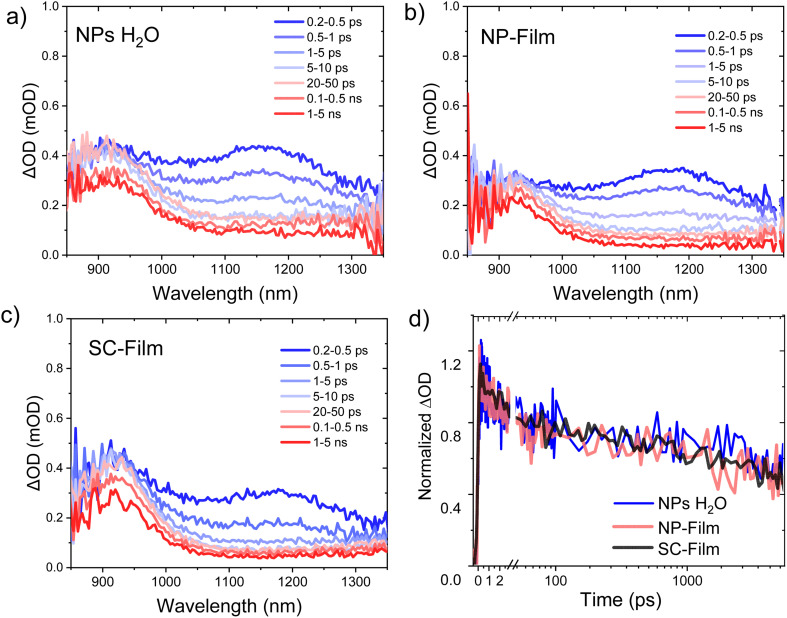
Ultrafast transient absorption spectra at different time delays of PM6 : PCBM 1 : 4 wt% in heterojunction (a) NPs dispersion in water, (b) NP-Film, and (c) SC-Film, excited at 550 nm and probed in the NIR (fluence: 5 μJ cm^−2^). (d) Comparison of transient absorption decay dynamics probed at 950 nm, assigned to PM6^+^ polaron decay.

We now turn to the decay dynamics of the PM6^+^ polarons generated by PM6 exciton separation. The uf-TAS spectra at longer (ns) decay times are dominated by a photoinduced absorption peaking at 950 nm, assigned to PM6^+^ polarons. Whilst exciton separation can also be expected to generate PCBM anions, its lower absorption coefficient of (6000 M^−1^ cm^−1^ at 1020 nm),^24^ prevent it from being readily detected. For all three heterojunction samples, the decay kinetics of PM6^+^ polarons extended beyond our timescale limit (>6 ns) and were fluence intensity dependent (Fig. S4†), indicative of the bimolecular recombination of separated charges. Indistinguishable decay kinetics were observed for this 950 nm feature for all three samples (Fig. 2d), indicative of similar bimolecular recombination kinetics on the ultrafast timescales addressed by these data, and again indicative of similar nanoscale molecular mixing of the PM6 and PCBM in all three samples.

### Charge recombination

**Fig. 3:**
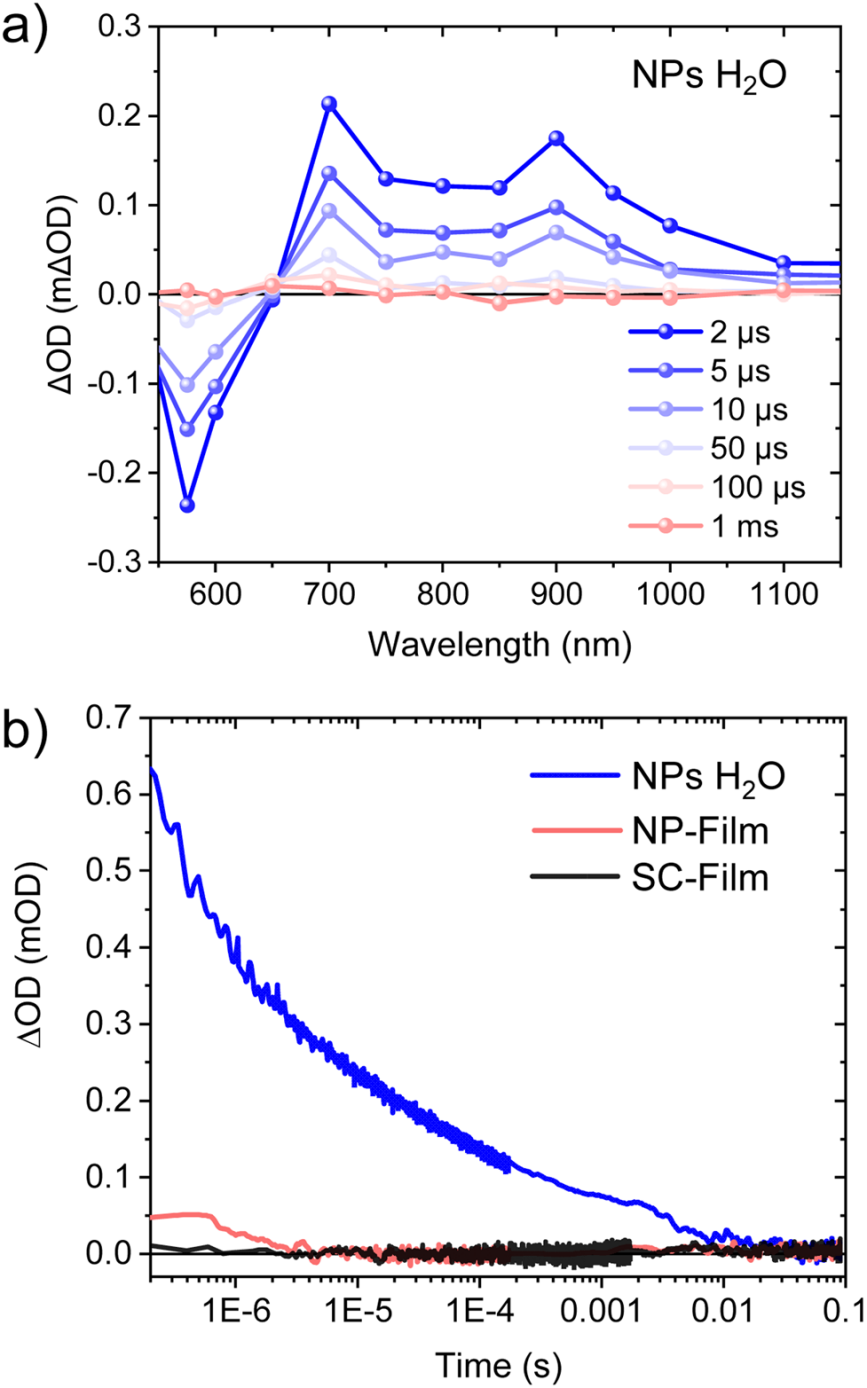
(a) Transient absorption spectra at different time delays of PM6 : PCBM 1 : 4 wt% NPs dispersed in water. (b) Comparison of transient absorption decay dynamics in heterojunction NP dispersion and films (adjusted at the same absorbed photons) probed at 700 nm, assigned to PM6^+^ polaron decay. Excited at 550 nm (fluence 220 μJ cm^−2^).

We turn now to explore the charge recombination dynamics in our three heterojunction samples at a longer microsecond to millisecond (μs to ms) timescale. Such slower timescale studies have previously been used to probe the trapping/de-trapping mediated recombination dynamics of spin-coated polymer : fullerene BHJ films.^25,26^Fig. 3 shows μs to ms TA spectra for our PM6 : PCBM NP dispersion, exhibiting a ground state bleaching (<650 nm) and a broad photoinduced absorption from 700 to 1100 nm (maximum at 700 nm and 900 nm), in agreement with our uf-TA spectra discussed above assigned to PM6^+^ species. These TA kinetics were not quenched in the presence of oxygen, which is consistent with this assignment and discounts potential contributions from PM6 triplet states (Fig. S5†). Control TA studies on a neat PM6 NP dispersion (Fig. S6†) showed two times lower amplitude and faster decay (within 5 μs) compared to the heterojunction NPs (probed at 700–1000 nm, Fig. S6†), consistent with the heterojunction resulting in a higher yield of separated charges, as indicated by our ultrafast data above. Most strikingly, the strong μs to ms TA signal observed for the PM6 : PCBM NP dispersion was strongly quenched for the NP-Film sample (15 fold smaller) and even smaller for the SC-Film sample, as shown in Fig. 3b (see also Fig. S7† for TA spectra of NP-Film). The absence of a significant μs to ms TA for the BHJ SC-Film is consistent with previous reports of the ns recombination kinetics for PM6-based BHJ films.^27^ It is striking that whilst all three heterojunction samples show similar kinetics on fs to ns timescales, their charge recombination kinetics on the μs to ms timescale are very distinct, with the NP dispersion showing a much larger and much longer lived decay (2 order of magnitude) than both NP- and SC-films, as we discuss further below.

**Fig. 4:**
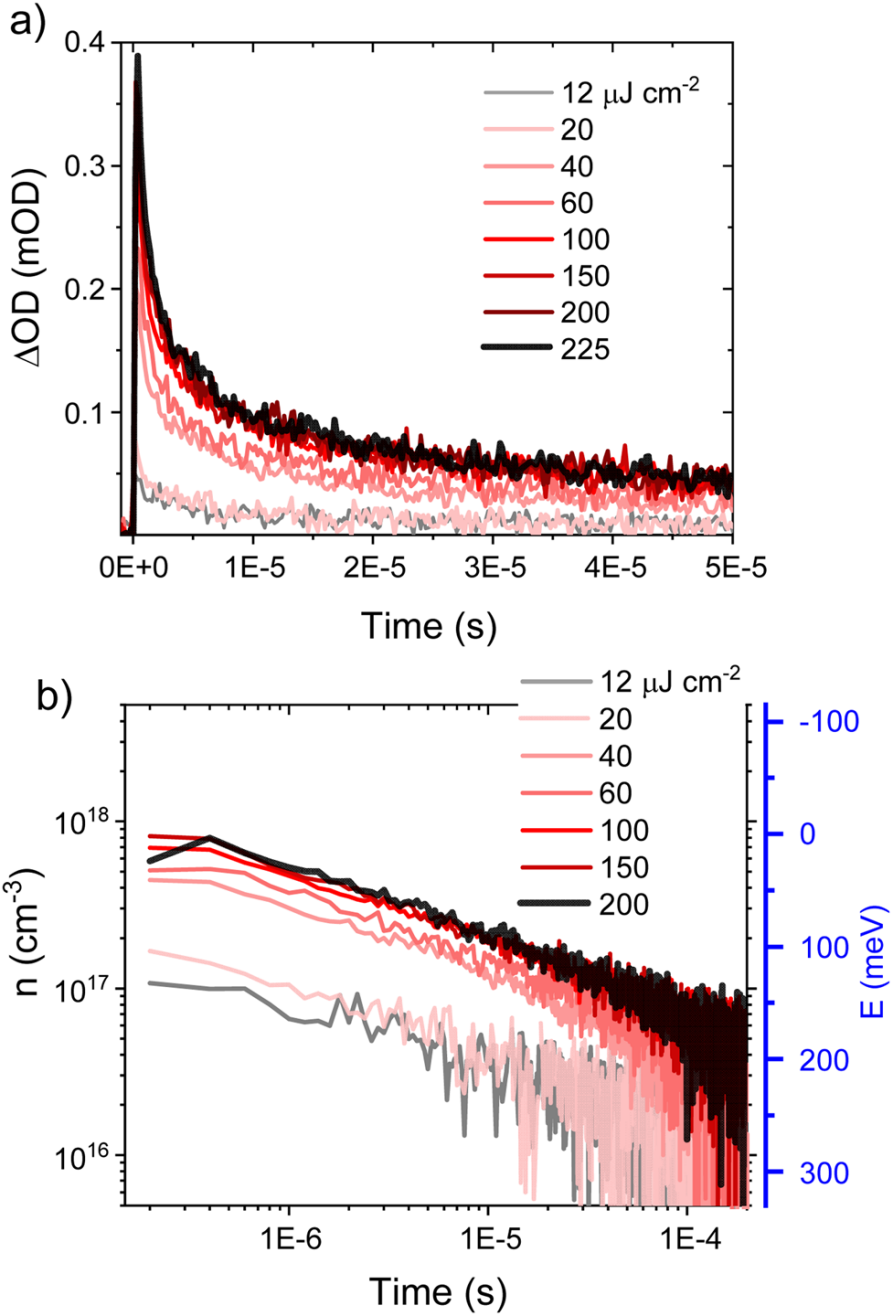
(a) Transient absorption decay dynamics of PM6 : PCBM 1 : 4 wt% NPs dispersed in water as a function of laser excitation density, probed at 700 nm. The excitation wavelength was 550 nm. (b) Charge carrier concentration *n*(*t*) calculated from TA kinetics shown in (a). Also shown on the right-hand *y*-axis (b) is the trap depth determined from these recombination kinetics for the highest (saturating) excitation density (see text for details).

We now turn to a more quantitative analysis of the slow charge recombination kinetics observed for the PM6 : PCBM NP dispersion. Fig. 4a shows the kinetics of the heterojunction NP dispersion probed at 700 nm at different fluence excitation densities (see kinetics probed at 900 nm in Fig. S8† showing analogous decay; therefore, 700 nm signal was analysed). Fig. 4b shows a log–log plot of these 700 nm data, with the Beer–Lambert law, used to convert the signal amplitude (ΔOD) into charge density, using a PM6^+^ molar extinction coefficient of 3.1 × 10^4^ M^−1^ cm^−1^ (see ESI† for details).^13^ It is apparent that these decay kinetics yield straight lines on this log/log plot corresponding to power law kinetics (ΔOD ∼ *t*^−*α*^), with gradients yielding an exponent *α* = 0.45 ± 0.02. It is also apparent that increasing the excitation fluence increases the amplitude of the TAS signal but does not change its decay kinetics. Such dispersive (*α* < 1) power-law kinetics are analogous to those reported previously in the OSC literature for polymer : fullerene blend films (for donor polymers such as PPV, MDM-PPV, P3HT and PDTSiTTz) and assigned to trapping/de-trapping mediated bimolecular charge recombination.^26,28^ More specifically, such dispersive power law behaviour has been attributed to an exponential tail of trap states, with the value of *α* being indicative of the energetic depth of these trap states, as we discuss further below. The amplitude of the TAS signal saturates at the highest excitation densities used at an initial charge density of ∼9 × 10^17^ cm^−3^ (*i.e.* PM6^+^ density at 400 ns for an excitation density of 150 μJ cm^−2^, equivalent to 5 × 10^13^ cm^−2^ absorbed photons), is indicative of saturation of available trap states. This is similar to the trap state density of 10^18^ cm^−3^ reported previously for P3HT : PCBM blends.^26,29^

### Charge accumulation under quasi-steady state irradiation

Finally, we measured the steady-state accumulation of PM6^+^ species under quasi-steady state irradiation, again comparing heterojunction NPs and films. In contrast to the relatively high intensity, but short, laser pulses used in the TAS studies above, we measure the photoinduced absorption (PIA) generated by low-intensity CW LED, selected to yield a similar flux of absorbed photons to one sun solar irradiation, and allow tracking of the kinetics of photogenerated charges in the millisecond to the second time range.^30^ Our previous PIA studies of PM6 : PCBM heterojunction NP dispersions showed the accumulation of remarkably long-lived PM6^+^ species under this quasi-steady state irradiation, corresponding to circa 600 PM6^+^ species per NP under 1 sun irradiation.^13^ Such charge accumulation was not observed in SC-Films prepared with the same materials. We focus herein on why these NPs are capable of accumulating such high densities of long-lived charges and how this observation correlates with our TAS studies.

**Fig. 5:**
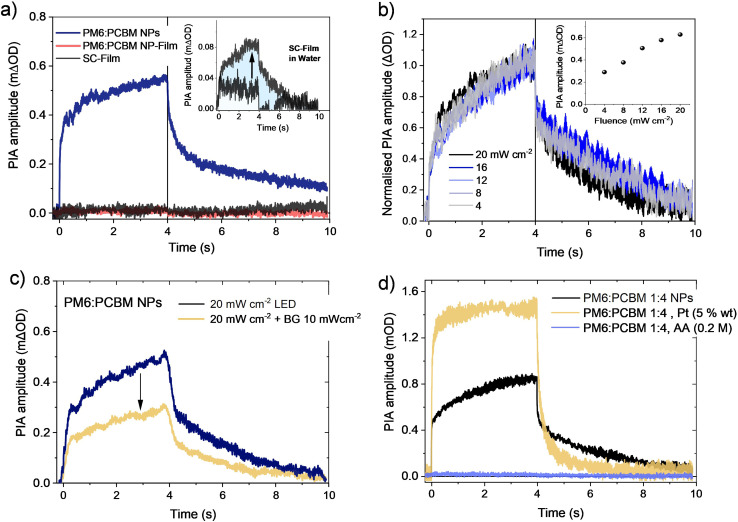
(a) Comparison of PIA kinetics of PM6 : PCBM 1 : 4 wt% heterojunction NP dispersions, NP-Film and SC-Film employing 1 sun equivalent, 4 s duration, LED excitation at 630 nm. Inset: PIA kinetics of 100 nm thickness SC-Film before (black) and after water immersion (grey trace). (b) Normalised PIA kinetics for the heterojunction NP dispersion as a function of LED fluence. Inset: PIA amplitude maximum as a function of LED fluence (mW cm^−2^). (c) PIA kinetics of heterojunction NP dispersion in the absence or under additional continuous LED background illumination (BG, 10 mW cm^−2^). (d) Comparison of the PIA kinetics of heterojunction NP dispersion, without and with the addition of ascorbic acid (AA, 0.2 M) or Pt (5 wt%). All data probed at 700 nm.

Fig. 5 shows typical PIA transients for the three samples studied herein, employing 4 second duration, 630 nm pulsed LED excitation (20 mW cm^−2^). The PIA spectra of heterojunction NP dispersion recorded at the maximum amplitude (4 s) after LED excitation are in good agreement with the μs TA spectra of PM6^+^ polaron reported above (compare Fig. S9† and 3a), with maximum absorption peaks at 700 and 900 nm, confirming the PIA signal also derives from PM6^+^ polaron absorption. The rise and decay kinetics of the NP dispersion PIA signal are both biphasic, with a fast phase with a 0.01 ms decay half-time and a slower phase extending over 10 seconds (Fig. 5). The quantum yields of accumulated PM6^+^ species were estimated from the measured ΔOD amplitudes and half-times of each decay phase as 3% for the fast phase decay and 0.04% for the slower decay (see details in ESI†). In contrast, both the NP-Film and SC-Film samples exhibited negligible PIA signals (Fig. 5a), consistent with the TA results described above (see Fig. 2). The absence of significant PM6^+^ PIA signal for the NP-Film indicates that large PM6^+^ accumulation observed for the NP dispersion is not specific to NPs, but rather is associated with their exposure to water, as we discuss in more detail below. Supporting this conclusion, the exposure of the BHJ SC-Film to water resulted in the appearance of an (albeit small, see inset Fig. 5a) PIA signal, indicative of water exposure inducing PM6^+^ accumulation.

Fig. 5b shows the fluence dependence of the PM6^+^ PIA signal at 700 nm for the NP dispersion. Additionally, the PIA kinetics were analysed under additional continuous background illumination (10 mW cm^−2^ of 630 nm LED), Fig. 5c. This background illumination was observed to reduce the PIA signal amplitude, consistent with the charge accumulation resulting from this background irradiation accelerating recombination kinetics and reducing the yield of long-lived PM6^+^ polarons. It is striking, however, that the long-lived polarons' decay kinetics are independent of both LED intensity (Fig. 5b) and background irradiation (Fig. 5c). Fig. 5d shows the NP dispersion PIA signal following the addition of either 0.2 M ascorbic acid (AA) or Pt (5 wt%), employed previously as respectively sacrificial electron donor and proton reduction catalyst for optimum photocatalytic performance.^13^ The addition of AA completely quenched the PIA signal, consistent with its efficient electron donation function (a similar quenching was observed in the presence of both AA and Pt). More strikingly, the addition of Pt resulted in an increase in PIA signal amplitude and removal of the slow phase of the rise/fall kinetics (with remaining a decay half-time of 0.02 s, Fig. 5d). This indicates that the addition of Pt increases the accumulation of long-lived PM6^+^ species, consistent with it extracting photogenerated electrons from the NP's to drive proton reduction to molecular H_2_.

## Discussion

In this work, we investigated the charge carrier dynamics in PM6 : PCBM 1 : 4 wt% heterojunction NP dispersions, dried NP films and films deposited by spin coating from an organic solution of the same materials. Both ultrafast transient absorption spectroscopy (Fig. 2) and photoluminescence (Fig. 1) data indicate efficient PM6 exciton separation within 1 ps timescale for all three samples, resulting in the generation of PM6^+^ and PCBM^−^ polarons (we note the low optical absorbance of PCBM^−^ prevents these species being observed directly). Our uf-TA kinetics indicate indistinguishable kinetics for the bimolecular recombination of these charges up to 6 ns for all three samples, assigned primarily to the recombination of untrapped charges. In contrast, TAS of residual charges on the slower μs to ms time shows very clear differences between the three samples, with the NP dispersion showing more than an order of magnitude larger signal, and slower recombination than the two film samples, with the SC-Film sample showing the smallest signal (Fig. 3). A similar trend was observed for the yield of accumulated PM6^+^ species under quasi-steady state irradiation, with only the NP dispersion exhibiting measurable PM6^+^ accumulation. This PM6^+^ accumulation is enhanced by Pt addition and quenched by AA, consistent with these long-lived PM6^+^ charges driving the photocatalytic activity of these heterojunction NPs. We turn now to discuss the origin of this striking retardation of charge recombination kinetics and increased long-lived PM6^+^ accumulation observed only for the PM6 : PCBM NP dispersion and not for either the NP-Film or SC-Film, considering 4 possible origins (i) the influence of the blends nanomorphology, (ii) the influence of NP surfactant, (iii) the influence of residual metal (palladium) clusters on charge recombination, and (iv) influence of the aqueous solvent environment on the NP dispersion.

### Influence of heterojunction nanomorphology

Our previously reported HRTEM images for PM6 : PCBM NP indicate a partial core/shell structure, with PM6 primarily in the shell.^13^ This contrasts with the BHJ morphologies typically observed for spin-coated donor/acceptor blends, such as the SC films studied herein. Our NP-films, fabricated by drop casting and drying the NP dispersion, can be expected to have similar heterojunction nanomorphologies to the NP dispersions. It is, therefore, striking that these NP-films exhibit similar optical signals on all timescales as the SC-films, clearly distinct (for the slower timescale measurements) from the data we obtain for the NP dispersion. This observation provides strong evidence that the remarkably long-lived charge accumulation we observe for NP dispersion does not result from a different heterojunction nanomorphology.

We note that all three samples exhibited similar ultrafast kinetics and PL quenching. This observation indicates similar nano/molecular scale mixing of PM6 and PCBM in all three samples, suggesting that the core and shell domains apparent for the NPs are most likely acceptor/donor rich rather than pure domains. However, a full morphological analysis is beyond the scope of this study and, moreover, not necessary as we have concluded that differences in nanomorphology cannot explain the differences in charge carrier kinetics we observe herein.

### Influence of the surfactant

The NPs employed in this study were fabricated using a mini-emulsion methodology, where a solution of the organic semiconductor(s) is processed into NPs by the formation of emulsion droplets in the presence of an ionic surfactant (TEBS for the study herein). Subsequent evaporation of the organic solvent (chloroform) results in the formation of an NP dispersion, with each NP stabilised in water by the surfactant. In heterojunction NPs, the surfactant influences the NP size and morphology by selective interaction with donor and acceptor blend components.^11^ The surfactant has also been shown to influence the photocatalytic activity in NP, affecting the amount of Pt loading on the NP surface and, consequently, the hydrogen production rate.^31^ However, our observation that long-lived charge accumulation is only observed for the NP dispersion and not for the NP-Film (which will retain the surfactant) indicates that the surfactant alone cannot be a main origin of the long-lived charge accumulation. Whilst we cannot rule out a synergistic effect of surfactant and water, or disruption of surfactant coating following film formation and drying, these data clearly point to the key role of the water environment in enabling long-lived charge accumulation.

### Residual metal clusters

In single organic polymer photocatalysts, it has been demonstrated that residual palladium (Pd) clusters from the polymer synthesis can act as co-catalysts for proton reduction and hydrogen evolution, therefore affecting charge separation and recombination in the organic semiconductors.^32^ This function requires the presence of water as a proton source. As such, it is possible that the enhanced long-lived charge accumulation observed in the presence of water (*i.e.*, the NP dispersion) results from enhanced Pd-mediated proton reduction, increasing the yield of residual PM6^+^ polarons (Fig. 5). In order to address this possibility, we also investigated a heterojunction NP dispersion prepared with purified PM6, achieved by washing a solution of PM6 with diethyldithiocarbamate, a palladium chelating agent, resulting in a decrease of the residual Pd from 600 ppm to 33 ppm (see details in ESI†). The PL spectra of neat purified PM6 NP showed negligible effect on PM6 exciton emission after purification, in agreement with invariant exciton decay in the uf-TA decay kinetics (Fig. S10†). Similarly, only minor differences were observed in the exciton and charge separation kinetics in the purified heterojunction NP dispersion (Fig. S11†). Crucially, the magnitude of the μs to ms TAS decay was unchanged by Pd purification, with only a minor change in decay kinetics (Fig. S12†). A similar trend was observed in the PM6^+^ accumulation, showing minor differences in the PIA kinetics decay after purification (Fig. S12†). It can thus be concluded that the much higher yield of PM6^+^ polarons observed for the normal (unpurified) heterojunction NP dispersion relative to the two dry films does not result from Pd-mediated proton reduction in water. We thus conclude that the enhanced yield of long-lived PM6^+^ species observed for the NP dispersion studied herein is not associated with the presence of residual Pd species, at least for the concentrations explored herein.

### Influence of the aqueous solvent environment

Having ruled out the effects of nanomorphology, surfactant and residual Pd as primary determinants of substantially increased yield of long-lived charges observed only for the NP dispersion, we turn to the consideration of the impact of the local aqueous environment surrounding each NP in this dispersion. The interplay between organic chromophores and the surrounding solvent has been widely studied in the literature.^33,34^ Solvation can strongly impact the photophysical properties of chromophores, leading to shifts in the energy of electronic states dependent on solvent polarity. In supermolecular systems, such as donor–acceptor dyads, higher solvent polarity has been correlated with higher charge separation yields, assigned to the stronger solvation of photogenerated charges increasing the reorganisation energy/energetic relaxation associated with charge generation.^35–37^ Similarly, in a study of a series of linear conjugated polymer photocatalysts, the polymer ionisation potential was calculated to shift with the solvent environment and correlated with photophysical and photocatalytic data.^38,39^ We, therefore, consider now in detail the possibility that enhanced long-lived charge generation in the NP dispersion may be associated with solvation/energetic stabilisation of these charges as they approach the organic/solvent interface.

In an early work, we demonstrated that the addition of glycol side chains to tuning the polymer IDTBT strongly impacted the photophysical properties in single and heterojunction NP dispersion. The addition of glycol side chains resulted in an increase in charge generation in pristine NPs, a lengthening of charge carrier lifetime in heterojunction NPs and an enhancement of the photocatalytic efficiency for hydrogen evolution.^40^ These effects were assigned to the glycol side chains, increasing water penetration into the bulk of the NPs and increasing the high and low-frequency dielectric response of the NP. However, for the heterojunction NPs fabricated with a hydrophobic PM6 polymer shell studied herein, the effect of water on the dielectric response inside the nanoparticle is probably negligible, and hence is likely to be most dominant as the photogenerated charges approach the NP surface – at the interphase with the polar surfactant.^41^ It thus appears most likely that the substantial increase in charge carrier lifetimes we observe for the heterojunction NPs results from an energetic stabilisation of these charges as they approach the NP surface due to increased water solvation. This will result in the localisation of PM6^+^ and PCBM^−^ species on, respectively, PM6 and PCBM-rich regions of the NP surface, resulting in a substantive spatial separation of the photogenerated charges and thus substantially suppressing their charge recombination kinetics.

This conclusion is supported by the observation that immersion of the BHJ SC-Film in water showed a 50% increase in PIA amplitude (measured at 4 s) and slower (ms to s) decay kinetics (see inset Fig. 5a). Although the resultant yield of charge accumulation was lower than that observed in the heterojunction NP dispersion, most likely due to limited water penetration into the hydrophobic film prepared by spin-coating,^38,42^ the increased yield of long-lived charges confirms that the presence of water suppresses charge recombination in our PM6 : PCBM system. We note that the increase in long-lived charge photoaccumulation with water exposure was removed by subsequent drying (*e.g.*: when forming NP-films), indicating this photoaccumulation does not result from structural or chemical defects caused by water exposure.^43,44^

### Quantification of the energetic loss associated with long-lived charge accumulation in heterojunction NP dispersions

As discussed above, we assign the long-lived charge accumulation observed for our NP dispersion to stabilisation of charges as they approach the NP surface due to increasing water (and potentially surfactant) polarisation. This stabilisation is energetically analogous to charge trapping, but functionally beneficial as it localises charges at the surface to drive ascorbic acid oxidation/proton reduction and also increases their lifetime. In order to quantify the magnitude of the energetic loss associated with this energetic stabilisation or ‘trapping’, we turn to previous analyses developed to analyse the charge trapping in organic thin films and solar cells.

The slow μs to ms TA charge recombination kinetics of the heterojunction NP dispersion studied herein exhibit dispersive power law decay kinetics, which, as discussed above, can be assigned to the trapping/de-trapping mediated recombination of charges in the presence of an exponential tail of shallow trap states.^25,26^ The characteristic energy *E*_ch_ of this exponential tail can be determined from the exponent *α* of the power law decay. For a tail state density of states *g*(*E*) ∝ exp(−*E*/*E*_ch_), it has been shown that *E*_ch_ can be determined from *E*_ch_ = *k*_B_*T*/*α*.^25^ A value of *α* ∼ 1, corresponding to ideal bimolecular recombination, indicates trap-free recombination, whilst a smaller value of *α* is indicative of more dispersive recombination and can be assigned to recombination in the presence of a wider energetic distribution of trap states. For example, in polymer fullerene OSCs, increases in film crystallinity induced by thermal annealing have been shown to influence *α* and, therefore, the trap distribution characteristic energy (ranging from 0.3 to 0.60).^26,45–48^

**Fig. 6:**
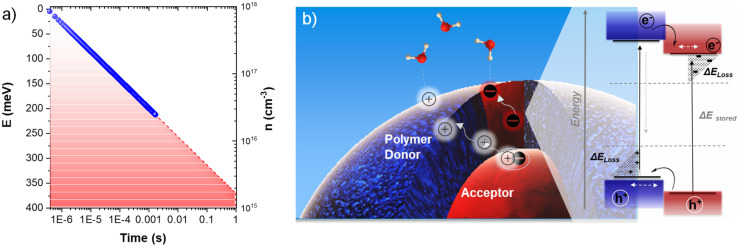
(a) Experimental energetic distribution of tail states in meV, calculated from TA kinetics as a function of charge lifetime. (b) Schematic illustration of a PM6 : PCBM 1 : 4 wt% heterojunction NP dispersion indicating the polymer polaron (hole) stabilisation and possible recombination pathways.

For the PM6 : PCBM heterojunction NP dispersion, our μs to ms TAS data yield a recombination power law coefficient *α* = 0.45, translating into a trap state characteristic energy *E*_ch_ = 55 meV. This characteristic energy is similar to those reported previously for dry P3HT : PCBM films (50–90 meV) but higher than those reported in the literature for PM6 : Y6 BHJ blend films, which have been reported to exhibit near-ideal (trap-free) behaviour.^21^ The saturation of the TA signal amplitude for high laser fluence can be assigned to filling all available traps. In this limit, the recombination kinetics directly track the trap depth, with the recombination kinetics becoming progressively slower as only more deeply trapped charges remain. As such, the kinetics of charge recombination as a function of charge density give an indication of the trap density of states. Fig. 6 shows a plot of *E*_trap_*versus n* obtained from the amplitude of the TA kinetic at 0.4 μs (see Fig. 4b), defining *E*_trap_ = 0 for *n* = 9 × 10^17^ cm^−3^ (*i.e.*, the initial charge density under saturating laser excitation). This trap energy is also plotted as the right-hand *y*-axis in Fig. 4b. It is apparent from these TAS data that the longest-lived charges live for >0.1 ms, corresponding to a trap depth of 150–200 meV (Fig. 6a).

The PIA decay kinetics obtained under quasi-steady state irradiation exhibit slower (1.9 s dominant decay half-time) decay than that observed in the μs to ms TA decay kinetics under pulsed laser excitation. Most simply, this can be attributed to the long-lived charges accumulating under continuous irradiation finding deeper trap sites, thereby enabling their spatial separation and increased carrier lifetime. As discussed above, this enhanced trapping or ‘localisation’ can be attributed primarily to the increased stabilisation of charges as they approach the NP surface and induce a polarisation of the surrounding water. Extrapolation of the trap depth *versus* charge lifetime plot obtained from our μs to ms TA data (Fig. 6a) to the 1 s decay dynamics, timescale measured in PIA, indicates an overall energetic loss of ∼350 meV for each charge. This additional energetic loss associated with charge relaxation upon exposure to water is illustrated in the diagram in Fig. 6b as the shaded grey areas.

The analysis above highlights that whilst the heterojunction NP aqueous dispersion exhibits a significant (2 order of magnitude) extension of charge carrier decay relative to the dry NP- and SC-Films, this lifetime extension comes at a significant energetic cost. We have previously highlighted the energetic loss associated with lifetime gain for a broad range of photovoltaic and photosynthetic/photocatalytic systems.^15^ Empirically, we observed an 88 meV energetic cost to gain *a* factor of 10 in lifetime across this broad range of solar conversion systems, slightly higher than the 59 meV minimal loss imposed by detailed balance.^15^ Our analysis indicates that the ∼1 × 10^6^ lifetime gain we observe for our aqueous NP dispersion results from an additional energetic loss of the charge pairs in the tale state resulting from charge relaxation due to water polarization, with the magnitude of this energetic loss being several hundred meV. Crucially, this charge stabilisation is associated with the localisation of these charges at PM6-rich/PCBM-rich regions of the NP surface for holes and electrons, respectively, facilitating subsequent reaction with ascorbic acid and charge transfer to Pt to drive proton reduction.

The energetic loss driving the generation of long-lived charges we observe herein for our aqueous heterojunction NP dispersion has direct parallels with strategies to increase carrier lifetimes in metal oxide photocatalysts. In our NP dispersion, this energetic loss/lifetime gain is driven by the large difference between the low frequency (<1 GHz) dielectric constant of the organic NP (*ε*_r_ ∼ 4) *versus* water (*ε*_r_ ∼ 80), indicative of a substantive difference in polarizability,^49^ which results in a substantial stabilisation of photogenerated charges as they approach the NP surface. This contrasts with metal oxides, which have similar, or indeed higher, dielectric constants than water. Instead, for metal oxides, charge stabilisation/lifetime gain at the semiconductor/electrolyte interface is typically driven by band bending resulting from a range of strategies, including, for example, externally applied bias, facet engineering and/or Schottky junction formation, all of which also result in an energetic loss associated with lifetime gain.^50–52^ The charge stabilisation we report herein is also analogous to that observed in molecular photochemistry, where an increase in solvent polarity can stabilise the generation of charges from initial photoexcitations.

Our PIA data indicate that the quantum yield of the long-lived charges we observe herein is significantly less than unity – approximately 3% for our NP dispersion, increasing to ∼6% following the addition of Pt. This is consistent with the promising but still modest EQEs we observe for photocatalytic H_2_ generation from this system.^13^ It appears likely that this quantum yield is primarily limited by the efficiency of transport of photogenerated charges to the NP surface, with most charges undergoing trapping and subsequent recombination in the NP bulk. This conclusion is consistent with our recent report of enhanced EQE for polymer NP with glycol side chains; such side chains can be expected to increase water penetration into the NP. In any case, it is apparent that increasing the quantum yield of charges with lifetimes long enough to enable charge transfer to the electrolyte whilst maximising the energy/reactivity of these charges are key challenges to enhancing the performance of organic nanoparticle photocatalysts.

## Conclusions

In this work, we combined transient and photo-induced optical spectroscopy to elucidate the differences in the charge carrier kinetics of D : A heterojunction NP dispersion and films. In both, the exciton separation in the D–A interface occurs on sub-ps timescale, resulting in indistinguishable bimolecular recombination of separated charges. In contrast, the NP dispersion showed notable slower recombination of photogenerated charges in a slower (millisecond-second) timescale. Such photogenerated charges in NP dispersion are even observed in quasi-steady state continuous light illumination conditions, with the photoinduced absorption kinetics reflecting the remarkable decay of active charges in seconds. Our results exclude the heterojunction nanomorphology, the surfactant and the residual metal (Pd) content as the leading source of the difference in charge recombination decay (∼2 order of magnitude) in NP dispersion and films, and suggest that the substantial increase in charge lifetime in NP dispersion is consistent with the energetic stabilization D^+^–A^−^ species on the heterojunction NP surface due water interaction.

The local charge-separated state stabilisation on the NP surface, which results in the lengthening of the charge carrier lifetimes to the ms time range, is sufficient to drive slow proton reduction under sacrificial conditions, as demonstrated by the differences in the yield of the accumulated charges after adding ascorbic acid and metal co-catalyst. However, the lifetime gains in the heterojunction NP dispersion (on the order of 10^6^), results in an additional energetic cost of circa 350 meV, possibly limited by the efficiency of transport of photogenerated charges to the NP surface, with most charges undergoing trapping and subsequent recombination in the NP bulk. A key challenge, therefore, is to develop heterojunction NPs with enhanced efficiency of charge localisation at the NP surface without increasing the energetic loss associated with this localisation. Addressing this challenge appears to be central to increasing the external quantum efficiencies of organic semiconductor photocatalysts for efficient visible light-driven solar-to-fuel conversion.

## Author contributions

S. G.-C. contributed to the investigation, spectroscopic measurements, formal analysis, and wrote the manuscript. J. K. synthesised the nanoparticle photocatalyst and contributed to the discussions. T. F. contributed to the spectroscopic measurements and analysis. J. R. D. corrected the manuscript. I. M and J. R. D. supervised the project. All authors discussed the results and commented on the manuscript.

## Conflicts of interest

There are no conflicts to declare.

## Supplementary Material

SC-015-D4SC04030A-s001

## Data Availability

The data supporting this article have been included as part of the ESI.†
