# Supplementary material for: Impact of water solvation on the charge carrier dynamics of organic heterojunction photocatalyst nanoparticle dispersions
Source: Chem Sci. 2024 Oct 21;15(45):19044–56. doi: 10.1039/d4sc04030a (PMC11514576; doi:10.1039/d4sc04030a)
Supplement: SC-015-D4SC04030A-s001 [file SC-015-D4SC04030A-s001.pdf]

Supplementary Information (SI) for Chemical Science.
This journal is © The Royal Society of Chemistry 2024

Impact of water solvation on the charge carrier dynamics of organic heterojunction photocatalyst nanoparticle dispersion

Soranyel Gonzalez-Carrero, [*a,b] Jan Kosco,[c,d] Teng Fei,[a] Iain McCulloch,[*c,d] James R. Durrant[*a,f]

[a] Department of Chemistry and Centre for Processable Electronics, Imperial College London, London W12 0BZ, United Kingdom.
[b] Institute of Molecular Science, University of Valencia, 46980 Paterna, Valencia, Spain
[c] King Abdullah University of Science and Technology (KAUST), KAUST Solar Center (KSC), Thuwal, 23955-6900, Saudi Arabia.
[d] Department of Chemistry, Chemistry Research Laboratory, University of Oxford, Oxford, OX1 3TA, United Kingdom
[e] Andlinger Center for Energy and the Environment, and Department of Electrical and Computer Engineering, Princeton University, Princeton, NJ, 08544, USA
[f] SPECIFIC IKC, College of Engineering, Swansea University, Bay Campus, Fabian Way, Swansea, Wales, SA1 8EN, United Kingdom.

Electronic Supplementary Information.

1. Materials.

Polymer PM6 was purchased from 1material. $PC_{71}BM$ was purchased from Ossila. Sodium 2-(3-thienyl)ethyloxybutylsulfonate (TEBS) was purchased from Solaris Chem. All chemicals were used without purification unless otherwise stated.

2. Synthesis of heterojunction nanoparticle. The colloidal donor: acceptor nanoparticles dispersion was prepared following our previously reported strategy.[1] Briefly, individual stock solutions (0.50 mg/mL) of PM6 and PCBM were prepared in chloroform and heated overnight (80 °C) to complete dissolution and filtered (0.2 um PTFE). Then, a nanoparticle precursor solution (5 mL) was prepared from the individual PM6 and PCBM stock solutions by mixing them in a ratio of 1:4 wt. % for PM6:PCBM. Subsequently, the nanoparticle precursor solution was added to a 0.5 wt.% solution of TEBS in MilliQ water (10 mL) and stirred vigorously for 15 min at 40 °C to form a pre-emulsion, which was then sonicated for 5 min with an ultrasonic processor (Sonics VibraCell VCX130PB) to form a mini-emulsion. Finally, the mini-emulsion was heated at 85 °C under an airflow to remove the chloroform, leaving a dispersion of NPs in water.

The size and morphology of the PM6:PCBM 1:4 wt. % NPs were previously characterised by combining dynamic light scattering and high-resolution transmission electron microscopy (cryo-HRTEM) to be 80 ± 20 nm and a core/shell morphology. [1] This NP size translates into an NP surface area of 20000 nm^2 (assuming spherical NP) and a total surface area of 1 mg of NP of 5.3 $\times 10^{16}$ nm^2 (considering an NP number of 2.67 ± 2.03 $\times 10^{12}$ for 1 mg of NP, see Nat Energy **2022**, 7, 340).[1]

3. Purification of polymer. The polymer PM6 was further purified by washing a solution of PM6 in chloroform with an aqueous solution of sodium diethyldithiocarbamate, as reported previously.[2] In brief, 50 mg of PM6 was dissolved in 60 mL of chloroform. After adding 100 mL water and 0.5 g sodium diethyldithiocarbamate, the two-phase mixture was stirred overnight at 50 C. The chloroform phase was washed 5 times with water, concentrated on a rotary evaporator, and precipitated into methanol. The concentration of residual palladium in the PM6 was quantified by ICP-OES. The purified polymer ($PM6_P$) was used to prepare heterojunction NP as described above.

4. Preparation of films. The PM6:PCBM (1:4 wt. %) SC-film was prepared by spin coating using an 8 mg ml-1 chlorobenzene solution at 2000 rpm and then annealing at 100 °C for 10 min. The film layer thickness was around 95 nm. The NP-film was prepared by drop casting the NP dispersion onto glass (2.2 cm^2) and drying under vacuum at room temperature.

5. Spectroscopic characterisation.

Absorption and photoluminescence spectra. The absorption spectra were recorded in an Agilent Cary 60 UV-Vis. The PL spectra of heterojunction nanoparticle dispersion were recorded using a Cary Eclipse fluorescence spectrometer.

Time-resolved photoluminescence decay kinetics were measured using the Time Correlated Single Photon Counting (TCSPC) system Deltaflex PPD900 (Horiba), using a 635 nm picosecond laser diode (1.0 mHz repetition rate) and a 710 nm detection wavelength corresponding to the emission peak of PM^ polymer. The TCSPC instrument response function is plotted in Figure S2.

Transient absorption spectroscopy (TAS). *Ultrafast TAS* analysis of heterojunction nanoparticles dispersed in water was carried out by using an amplified Ti:sapphire laser (Solstice, Spectra-Physics) with an 800 nm laser pulse (< 200 fs, 1 kHz repetition rate). Using a beam splitter, the laser pulse is then divided into the pump and the probe. An optical parametric amplifier (TOPAS Prime, Light Conversion) and a frequency mixer (NirUVis, Light Conversion) generate the pump laser at the excitation wavelength. The probe pulse at specific time delays is generated through a mechanical delay stage, which delays it by an adjustable period (maximum of 6 ns) relative to the pump pulse. The continuous white light probe in the visible (450−800 nm) or NIR (850−1400 nm) region is generated by focusing the probe pulse into a sapphire crystal. Then, the probe pulse is divided before the sample into two pulses; one is directed to the sample, and the other is used as the reference. Both pulses are directed to a separate multichannel spectrometer (Si or InGaAs sensor). The continuum probe pulse on

the samples is spatially overlapped with the pump pulse. The pump pulse is chopped by a synchronised chopper with a frequency of 500 Hz. Pulse energies were measured using an energy meter (OPHIR Photonics, VEGA P/N 7Z01560) with a 500 μm diameter aperture. The samples were measured in the Argon atmosphere, including the films. All NP dispersion was measured at equal numbers of absorbed photons, with an absorbance of 0.6 at the excitation wavelength.

Microsecond−Second TAS was carried out by using a Nd:YAG laser (OPOTEK Opolette 355 II, 6 ns pulse width), which generates UV pulses (355 nm) or visible/NIR pulses (410−2200 nm). The probe beam is generated from a 100 W quartz halogen lamp and sequentially directed through the sample and then to a monochromator, which is directed onto a Si photodiode detector (Hamamatsu S1722-01). Pump pulses are directed to the sample through a liquid light guide and are overlapped with the probe beam at the position of the sample. Data acquisitions are triggered by scattered laser light using a photodiode. Appropriate long-pass filters are positioned before the sample and between the sample and the detector to attenuate scattered laser light. A home-built LabVIEW-based software package was used to acquire the data on two different time scales simultaneously: the microsecond−millisecond signal is sampled using an oscilloscope (Tektronix DPO 3012) after amplification (Costronics 2011 amplifier), whereas the millisecond−second signal is sampled without amplification using a DAQ card (National Instruments USB-6361). Pulse energies were measured using an energy meter (OPHIR Photonics, VEGA P/N 7Z01560). All NPs dispersions in water were prepared at equal numbers of absorbed photons, with an absorbance of 0.6 at the excitation wavelength and measured under an argon atmosphere.

Charge density calculation from TA results. The experimental change in transient absorbance (ΔOD) has been converted to charge concentration density n (in units of cm^{-3}, assuming charge neutrality $n=p$) using the Beer-Lambert law, according to equation 1.

$$n = \frac{N_A \, \Delta OD}{\varepsilon \, d} \quad (1)$$

Where N_A is Avogadro's number, d is the estimated sample thickness (8×10^{-6} cm), and ε is the estimated molar extinction coefficient for photogenerated polarons (3.1×10^4 Mcm^{-1}).[1] At an excitation density of 150 μJ cm^{-2} this corresponds to 9×10^{17} cm^{-3} $PM6^+$ density at 400 ns.

Photoinduced Absorption Spectroscopy (PIAS). The PIAS analysis was carried out on a modified microsecond−second TAS setup. A high-power LED (Cree XLamp XP-E XPERED-L1-0000-00801, 630 nm) was used as an excitation source driven by a high-precision DC power supply (TTi QL564P). The LED is directed to the sample through a liquid light guide. Light pulses are generated via a MOSFET transistor (STMicroelectronics STF8NM50N), and the gate is modulated by the DAQ card

(National Instruments, USB-6361). All data were sampled without prior amplification using the DAQ card. Excitation fluences were measured with a digital power meter (Thorlabs PM100) using a silicon photodiode power sensor (Thorlabs S120UV).
All the PIA kinetics were measured in transmission mode at the same conditions: 630 nm LED excitation and fluence 20 mW cm^{-2}. The NP dispersions were prepared with equal numbers of absorbed photons (absorbance of 0.6 at the excitation wavelength) and measured in an argon atmosphere.

Quantum yield of charge generation from PIA. The quantum yield (*QY*) of photogenerated charges (holes) in the heterojunction NP dispersion in water probed in PIA (Fig. 5) was calculated by the ratio between the moles of the photogenerated generated holes to the absorbed photon flux multiplied by the charge lifetime (half lifetime, $t_{50\%}$), equation 2:

$$QY = \frac{moles\ of\ accumulated\ photogenerated\ holes}{moles\ of\ absorbed\ photons\ \ \times t_{50\%}} \times 100 \tag{2}$$

Where, the *moles of accumulated photogenerated holes* was estimated by the Beer-Lambert law using the measured ΔOD amplitude in the PIA kinetics (ΔOD amplitude for the fast and slow phase, the Fig. 5). The $PM6^+$ extinction coefficient ε at λ = 700 nm was estimated to be 31,500 M^{-1} cm^{-1} using the initial ΔOD amplitude of uf-TA spectra of PM6 NP dispersion at the lowest excitation density conditions (see Nat Energy **2022**, 7, 340) and assuming a unity charge generation yield per absorbed photon. [1] The *absorbed photons* were calculated from the incident 635 nm LED photon flux and the steady-state absorbance of the NP dispersion (0.6 absorption at the excitation wavelength).

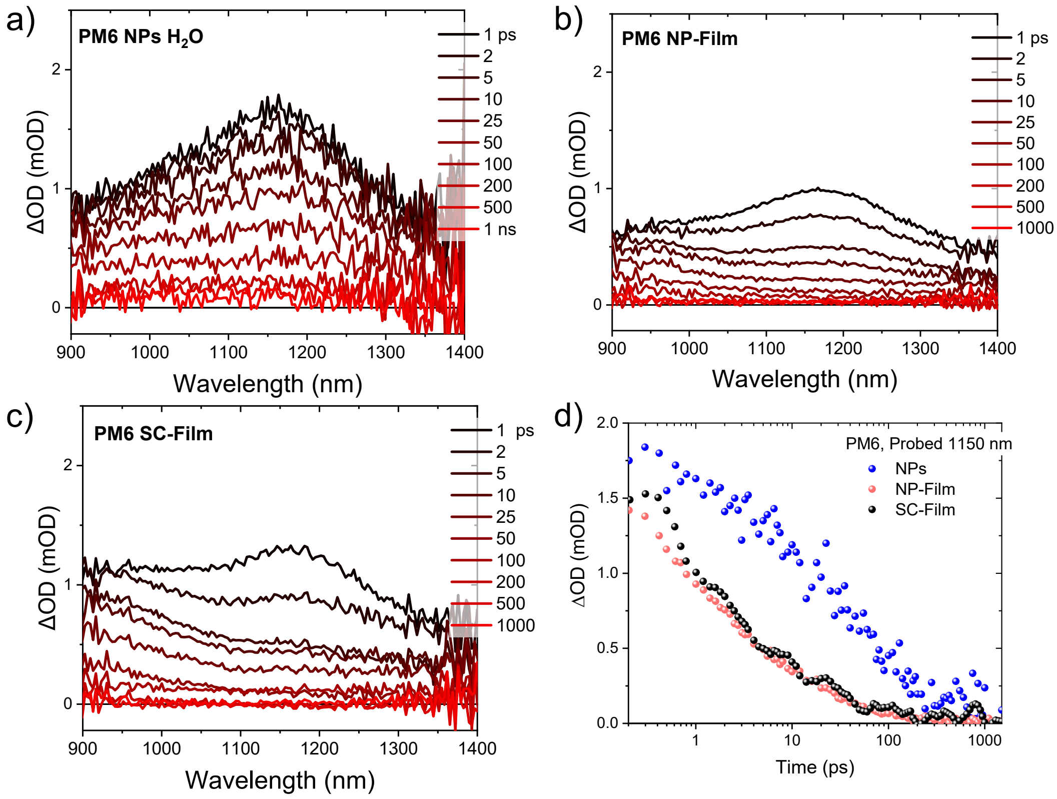

Figure S 1. Ultrafast transient absorption spectra at different time delays of a) PM6 NPs dispersion in water, b) PM6 NP-Film, and c) PM6 SC-films, probed in the NIR. d) Comparison of transient absorption decay dynamics for neat PM6 NPs, NPs solid film and SC-films probed at 1150 nm, assigned to PM6 exciton decay. Excitation at 550 nm (fluence: 5 μJ cm^{-2}). We note these decay dynamics are significantly faster than those observed by time-correlating single photon counting (**Fig. S 2** below), attributed to the impact of exciton-exciton annihilation (EEA). The faster exciton decay observed for BHJ SC-Film and NP-Films relative to isolated NPs in water dispersion (Figure S1d) is most likely due to faster non-radiative decay to ground, although it is also possible that exciton-exciton annihilation (EEA) is faster in the heterojunction films relative to isolated NPs in water dispersion.

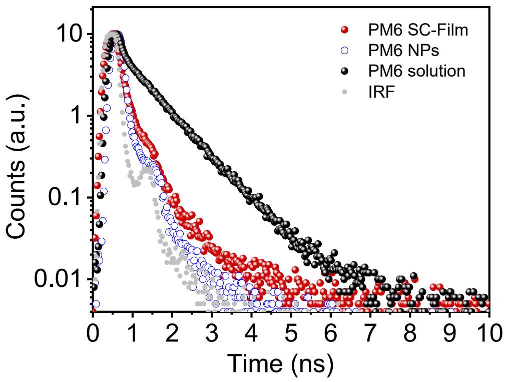

Figure S 2 Comparison of PL emission decays of neat PM6 in SC-Films, NPs dispersion in water, and PM6 solution (in chloroform), showing faster PM6 exciton decay in films and NP dispersion. The PL decay kinetics were recorded using a time-correlating single photon counting system upon a 635 nm LED excitation pump and probed at 710 nm. The PL decay kinetic of PM6 in solution was adjusted to mono-exponential decay, yielding a lifetime of 1.4 ns.

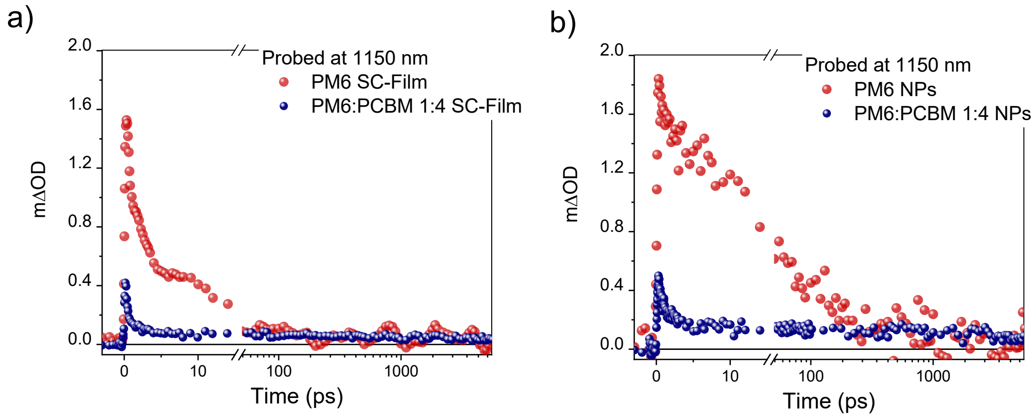

Figure S 3. Comparison of transient absorption decay dynamics of a) neat PM6 and PM6:PCBM 1:4 SC-Film, and b) neat PM6 NP and PM6:PCBM 1:4 NPs dispersion; excited at 550 nm (fluence: 5 µJ cm^{-2}) and probed at 1150 nm, signal assigned to PM6 exciton decay. The heterojunction probed at 1150 nm exhibited faster (up to 25 times) of PM6 decay, with a residual long-lived signal in the PM6:PCBM 1:4 heterojunction NPs dispersion and PM6:PCBM 1:4 SC-Film, assigned to long-lived PM6 polarons (> 6 ns), indicating efficient PM6 exciton separation within 1 ps timescale in the heterojunctions.

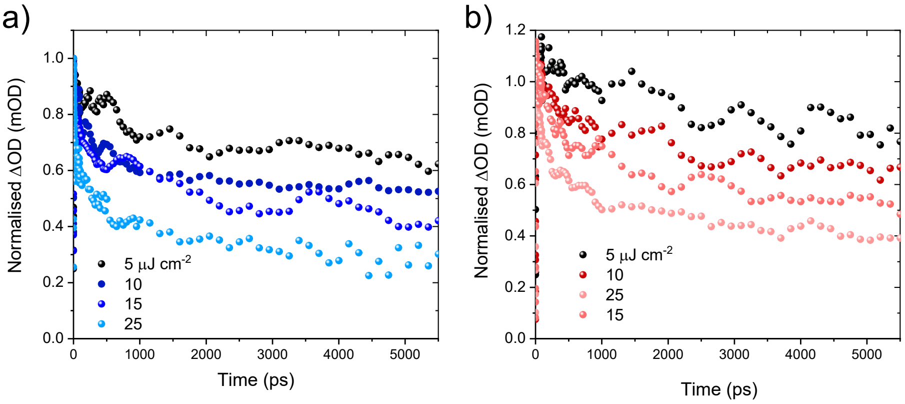

Figure S 4. Normalised transient absorption kinetics as a function of excitation density of a) PM6:PCBM 1:4 NPs dispersion in water and b) PM6:PCBM 1:4 SC-Film, excited at 550 nm and probed at 950 nm, assigned to $PM6^{+}$-$PCBM^{-}$ bimolecular recombination.

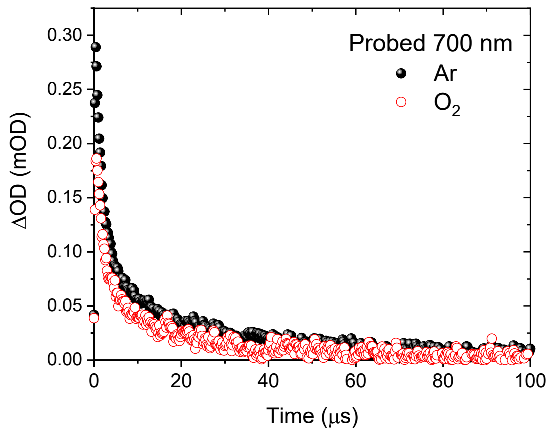

Figure S 5. Transient absorption decay of PM6:PCBM 1:4 NPs dispersion in water probed at 700 nm under argon (black) and oxygen (red) atmosphere. Excitation at 550 nm (fluence: 200 μJ cm^{-2}).

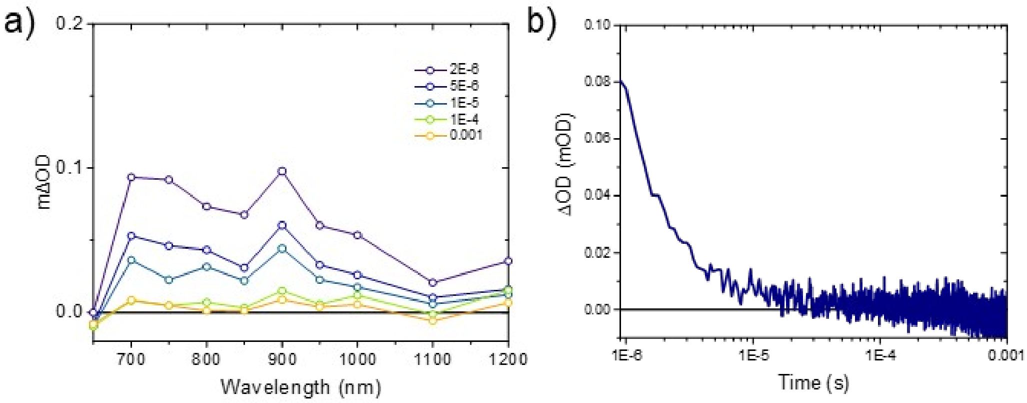

Figure S 6. a) Transient absorption spectra at different time delays of PM6 NPs dispersion in water. b) Transient absorption decay of PM6 NPs probed at 700 nm. Excitation at 550 nm (fluence: 200 μJ cm^{-2}). Under these excitation conditions, the transient spectrum of PM6 exhibited similar absorption bands to those observed for PM6:PCBM 1:4 *wt. %.* heterojunction NPs (Fig. 3), assigned primarily to PM6^{+}. This charge generation in neat NPs is most likely resulting from water-induced charge stabilisation. However, the fast decay (within 2 μs) indicates only a limited spatial separation of these charges relative to the heterojunction NPs.

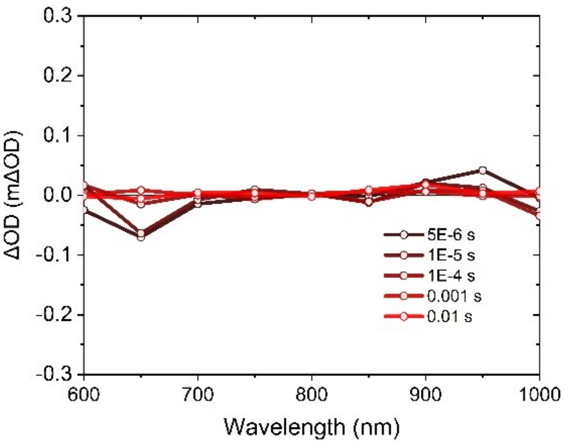

Figure S 7. Transient absorption spectra at different time delays of PM6:PCBM 1:4 NP-Film, showing the fast (μs) decay. Excitation at 550 nm (fluence: 200 μJ cm^{-2}).

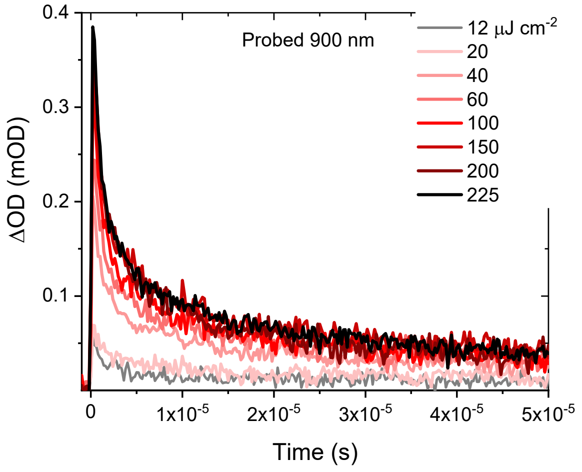

Figure S 8. a) Transient absorption decay dynamics of PM6:PCBM 1:4 NPs dispersion probed at 900 nm as a function of laser excitation density, showing similar decay as observed in the kinetics probed at 700 nm. The excitation wavelength was 550 nm.

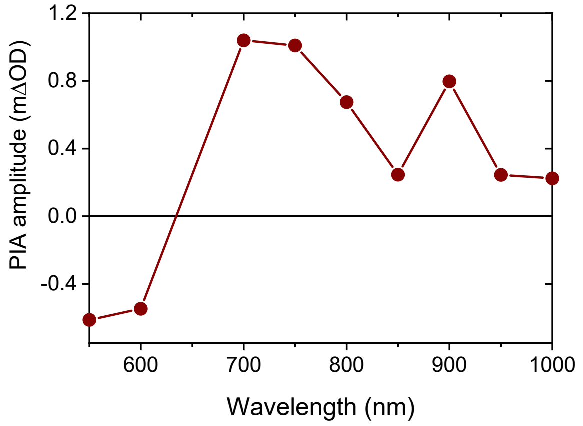

Figure S 9. a) Photoinduced absorption spectra of PM6:PCBM 1:4 heterojunction NPs dispersion measured at 4s. LED excitation (630 nm, 20 mW cm^{-2}) during 4 s.

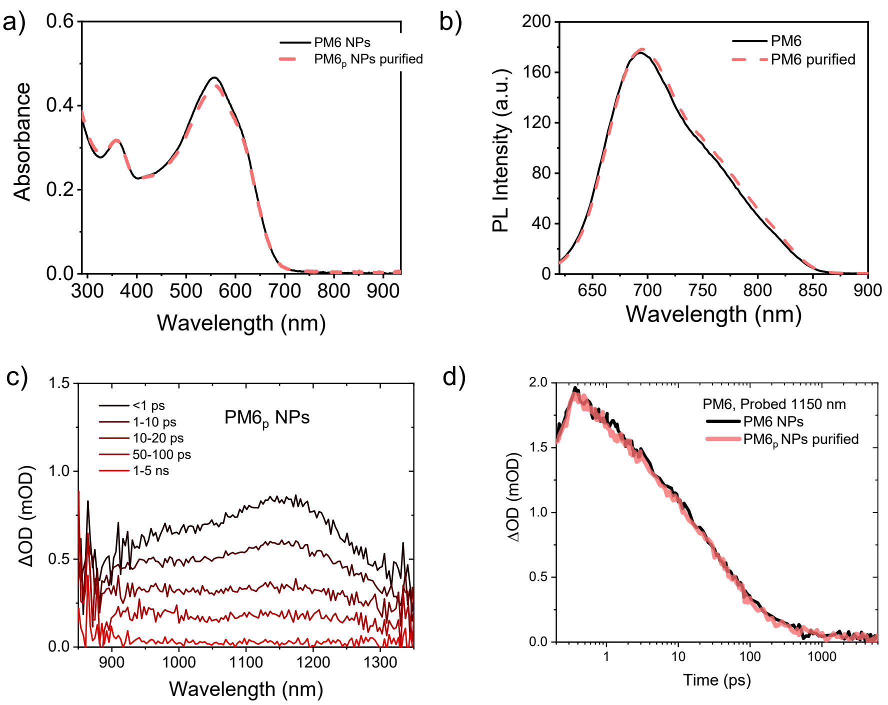

Figure S 10. a,b) Absorption and steady-state PL spectra of PM6 NPs dispersion in water before and after purification (*PM6p*, red trace); PL measured upon 550 nm excitation wavelength. c) Ultrafast TA spectra of purified $PM6_p$ NP dispersion at different time delays, showing PM6 exciton absorption. d) Comparison of the transient absorption decay of PM6 and purified $PM6_p$ NPs dispersion probed at 1150 nm, assigned to PM6 exciton decay.

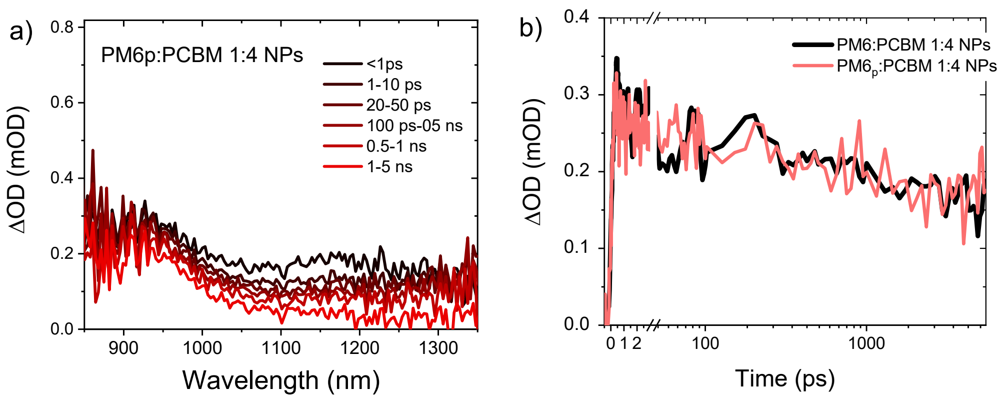

Figure S 11 a) Ultrafast TA spectra of purified $PM6_P$:PCBM 1:4 heterojunction NPs dispersion, showing polaron absorption (centre at 950 nm). The NP dispersions were excited at 550 nm (5 μJ cm^{-2}) and probed in the NIR. b) Comparison of transient absorption decay dynamics for purified $PM6_p$:PCBM 1:4 and PM6:PCBM 1:4 heterojunction NPs dispersion, probed at 950 nm, assigned to $PM6^+$ polaron decay. Excitation at 550 nm (fluence: 5 μJ cm^{-2}).

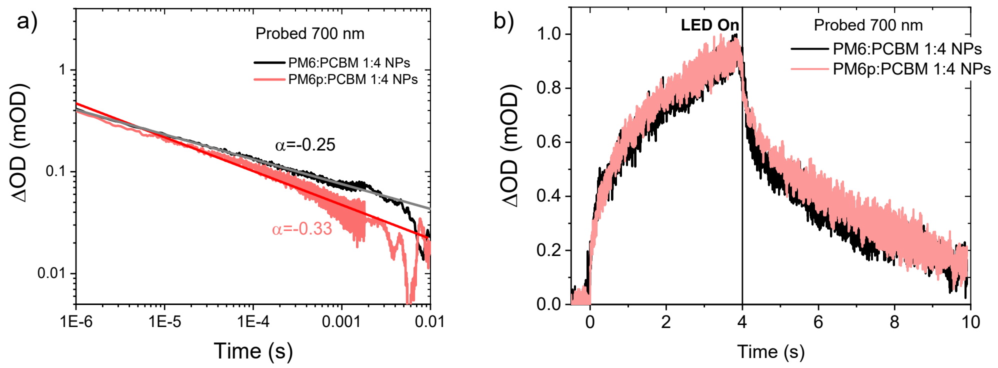

Figure S 12. a) Comparison of TA kinetics of PM6:PCBM 1:4 and purified $PM6_P$:PCBM 1:4 heterojunction NPs dispersions in water, probed at 700 nm and adjusted at the same amount of absorbed photons. Excitation at 550 nm (fluence: 200 μJ cm^{-2}). b) Normalised PIAS kinetics of PM6:PCBM 1:4 and purified $PM6_P$:PCBM 1:4 NPs dispersion in water, probed at 700 nm. LED excitation at 630 nm (20 mW cm^{-2}).

REFERENCES

(1) Kosco, J.; Gonzalez-Carrero, S.; Howells, C. T.; Fei, T.; Dong, Y.; Sougrat, R.; Harrison, G. T.; Firdaus, Y.; Sheelamanthula, R.; Purushothaman, B.; Moruzzi, F.; Xu, W.; Zhao, L.; Basu, A.; De Wolf, S.; Anthopoulos, T. D.; Durrant, J. R.; McCulloch, I. Generation of Long-Lived Charges in Organic Semiconductor Heterojunction Nanoparticles for Efficient Photocatalytic Hydrogen Evolution. *Nat Energy* **2022**, *7* (4), 340–351. https://doi.org/10.1038/s41560-022-00990-2.

(2) Kosco, J.; McCulloch, I. Residual Pd Enables Photocatalytic H2 Evolution from Conjugated Polymers. *ACS Energy Lett.* **2018**, *3* (11), 2846–2850. https://doi.org/10.1021/acsenergylett.8b01853.
